# Rational design and *in vitro* testing of new urease inhibitors to prevent urinary catheter blockage†

**DOI:** 10.1039/d4md00378k

**Published:** 2024-09-12

**Authors:** Rachel A. Heylen, Nicola Cusick, Tom White, Emily J. Owen, Bethany L. Patenall, Martin Alm, Peter Thomsen, Maisem Laabei, A. Toby A. Jenkins

**Affiliations:** a Department of Chemistry, University of Bath Bath BA2 7AY UK a.t.a.jenkins@bath.ac.uk; b School of Cellular and Molecular Medicine, University of Bristol BS8 1TD Bristol UK; c Biomodics ApS Fjeldhammervej 15 2610 Rødovre Denmark

## Abstract

Catheter associated urinary tract infections (CAUTI) caused by urease-positive organisms can lead to catheter blockage: urease metabolizes urea in urine to ammonia causing an increase in pH and hence precipitation of struvite and apatite salts into the catheter lumen and bladder leading to blockage. Acetohydroxamic acid (AHA) is the only urease inhibitor currently approved for patient use, however, it is rarely used owing to its side effects. Here, we report the identification and development of new urease inhibitors discovered using a rational *in silico* drug design approach. A series of compounds were designed, the compounds were screened and filtered to identify three compounds which were tested in *in vitro* urease activity assays. *N*,*N*′-Bis(3-pyridinylmethyl)thiourea (Bis-TU) outperformed AHA in activity assays and was tested in an *in vitro* bladder model, where it significantly extended the lifetime of the catheter compared to AHA. Bis-TU was delivered *via* a diffusible balloon catheter directly to the site of activity, thus demonstrating localized drug delivery. This cost-effective drug design approach allowed the identification of a potent urease inhibitor, which could be improved through iterative repeats of the method, and the process of design could be utilized to target other diseases.

## Abstract 2

*In silico* identification of urease inhibitors based on thiourea, tested to determine IC_50_ and tested on a catheterised *in vitro* bladder model, showing efficacy in reducing catheter blockage.

## Graphical abstract

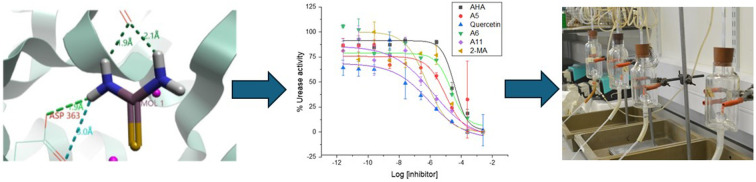

## Introduction

Catheter-associated urinary tract infections (CAUTI) are widely recognized as a consequence of using a long-term indwelling urinary catheter (Foley).^1^ After 4 weeks, over 90% of catheter users are likely to have bacteriuria (bacteria present in the urine).^1^ As a result, CAUTI increases morbidity and economic burden; in the UK, CAUTI accounted for 45 717 excess NHS bed days between 2016–2017 and costs the NHS £1.50–2.25 billion per year.^2–4^ Globally, UTIs affect approximately 150–250 million patients per year.^5^ The catheter increases the risk of infection because it bypasses the bladder's natural ability to fill and void urine, therefore causing a residual pool of urine in the bladder around the tip of the catheter and the balloon. This pool of urine provides nutrients for bacterial colonization, thus increasing the likelihood of CAUTI.^6^ The Foley catheter is the most commonly prescribed prosthetic medical device.^6^ In patients, asymptomatic bacterial colonization is common; approximately 12.5% of patients develop symptomatic CAUTI, and given the high number of catheter users, this results in significant morbidity globally.^7,8^ Around 1 in 3 long-term urinary catheter users experience at least one, and often many, urinary catheter blockage every year; the majority of these blockages are due to urease-positive infections.^7,9^ Urease, the virulence factor associated with catheter blockage, metabolizes urea to ammonia and carbonic acid.^10^ This liberation of ammonia increases the pH within the bladder, causing precipitation of urinary salts, apatite and struvite, thus leading to the formation of crystals.^11^ Encrustations form on and around the catheter, resulting in occlusion of the catheter.^11^*Proteus mirabilis* is the most common urease-positive microorganism associated with catheter blockage, and is found in 80% of blocked catheters.^7^ A blocked urinary catheter causes painful distention of the bladder, and forces the urine up the ureters towards the kidneys, thus increasing the risk of pyelonephritis.^12^ Consequently, a blocked catheter increases the risk of a bloodstream infection, urosepsis, and ultimately is associated with increased mortality.^12,13^

Ideally, a patient using a long-term Foley catheter will be well assessed and the ‘pattern of catheter life’ recorded by community nurses or at the clinic. This assessment, in theory, is used to track the catheter's performance and therefore ensure replacement of the catheter prior to complications.^14^ In reality, this does not always occur and, rather than a planned catheter removal, catheters often have to be removed when blocked, this is often referred to as ‘crisis care’.^15^ A blocked catheter is generally treated by one of two methods, either complete removal of the catheter and re-insertion of a new catheter, or a bladder washout is performed. Removal of the catheter is often painful for patients; encrustations anchor the catheter within the bladder and removal can damage the urethra. In serious cases catheters have to be surgically removed.^11,13^ Bladder washout is a general term used for three types of treatment: bladder irrigation, bladder washouts, and bladder instillations. Overall, the aim of this treatment is to remove the encrustations from the bladder and therefore extend the lifetime of the catheter.^14^ The solutions used for washouts can vary from sterile saline to citric acid solutions which aim to decrease the pH of the bladder to dissolve the encrustations and prevent further blockage. Evidence for the efficacy of these treatments is lacking, and some reports claim that the solutions damage the mucosa lining of the bladder.^13,14^ Alternative care pathways, such as prescription of antibiotics (prophylactic or treatment), are generally discouraged, owing to risks of developing resistant bacteria.^13,16^ A long-term catheter is often colonized by more than one species of bacteria, therefore antibiotic prescription should be carefully controlled due to the increased risk of developing antimicrobial resistance in multiple bacterial species.^17^ However, in many clinics, antibiotics are still prescribed for long-term catheter users.^13^ As described in a recent *Lancet* review, antibiotic resistant infection was determined as a leading cause of death worldwide; this is an additional challenge for treating CAUTI.^18^ If possible, antibiotic treatment should be avoided to prevent resistance occuring.^1^ Alternative treatments for catheter blockage are: emergency catheter change or catheter maintenance by nurses.^13^ If the catheter blockage is not treated quickly enough then the patient's morbidity increases, and the likelihood of hospitalization increases.^19^

Alternative treatments to catheter changes, washouts, and antibiotics are thus of great interest. One such therapy uses urease inhibitors. Urease inhibitors act as an anti-virulence treatment, disarming the virulence factor of blockage, urease. The concept is such that urease inhibitors do not kill the bacteria, unlike antibiotics, but disarm the ability of the bacteria to metabolize urease, therefore there is no elevation in pH, no formation of crystalline biofilm and no catheter blockage.^20^ Acetohydroxamic acid (AHA) is the only registered urease inhibitor; it has been used to treat recurrent catheter blockages and the formation of bladder stones, as well as to treat hyperammonemia caused by *Helicobacter pylori* infections in the stomach.^21–23^ AHA has been registered in the USA, under the name Lithostat; and in Kuwait and Spain under the name Uronefrex.^21,24,25^ However, AHA exhibits toxic side effects including hemolytic anemia and teratogenesis (birth defects formed in an embryo/fetus).^26^ Therefore, the use of AHA in the clinic has decreased and it has been withdrawn from the general market.^22,24^ Other urease inhibitors have been explored, not just as a treatment for urease-positive bacterial infections, but also for use in agriculture to prevent nitrogen loss by ammonia volatilization.^27^ These identified urease inhibitors have been well reviewed by Rego *et al.*, Kafarski and Talma, and Kappaun *et al.*^24,28,29^

In this study, we introduce an alternative methodology to identify new urease inhibitors. We propose the use of *in silico* docking experiments to examine the interaction of known inhibitors with urease, followed by virtual screens to identify new potential inhibitors. These inhibitors can then be sourced/synthesized for *in vitro* testing against urease. This method has been described as rational drug design because the screen is designed based on known inhibitors and decision points throughout the process. This is different to a traditional drug discovery approach where generally millions of random compounds are screened against a target. The hypothesis is this molecular modelling approach will yield a series of new potent inhibitors without the laborious physical synthesis and screening of many compounds.

Another important element of blockage treatment is the delivery of the drug to the target site. If administered systemically, the drug must be excreted in the urine into the bladder at a therapeutic level, but achieving this level can lead to additional side effects and the drugs can be metabolized in the liver.^30^ Intravesical drug delivery systems deliver the active pharmaceutical ingredient (API) directly into the bladder, where it is often used in the treatment of bladder cancer and is the principle behind bladder washouts.^30^ Biomodics ApS, Denmark, have developed a novel drug delivery catheter, where the drug is delivered through the catheter balloon, which is composed of an interpenetrating polymer network (IPN). A hydrophilic poly(2-hydroxyethyl methacrylate-*co*-poly(ethylene glycol) methyl ether acrylate) (poly(HEMA-*co*-PEGMEA)) network was integrated into the silicone elastomer of the catheter balloon. This formation of an IPN makes the balloon permeable, so it can be filled with a treatment solution, allowing sustained delivery into the bladder. This has been shown to be effective against *Escherichia coli* CAUTI and treatment of bladder cancer using a porcine model.^31,32^ Here we show the identification of a new urease inhibitor, identified by rational *in silico* drug design and subsequent validation through *in vitro* experiments. This inhibitor is more potent than the industrial standard, AHA, and can be delivered directly into the bladder using Biomodics' IPN catheter.

## Results and discussion

### *In silico* docking results

**Fig. 1:**
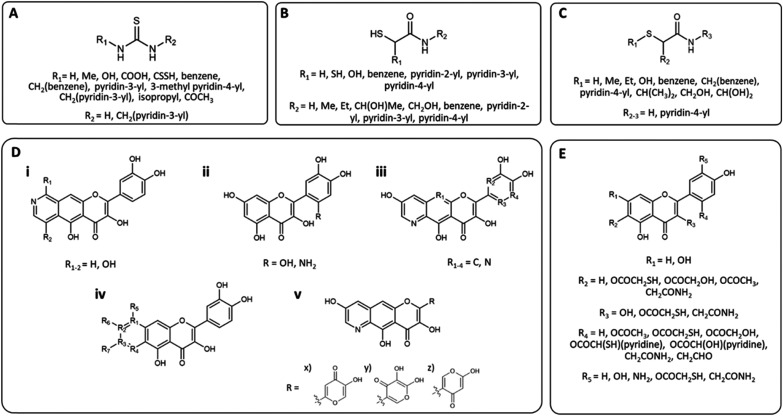
Chemical structures of the series of compounds docked. Series (A) based around thiourea, (B) and (C) 2-MA, (D) quercetin, and (E) 2-MA and quercetin. Full structures can be found in Fig. S3,† compounds are drawn using ChemDraw 19.1.1.21 (PerkinElmer Informatics, Waltham, Massachusetts, US).

The compound screen was designed based on known urease inhibitors, allowing for an informed drug design process. *In silico* docking experiments were performed using a high resolution crystal structure of urease from *Sporosarcina pasteurii* (Protein Data Bank (PDB) = 4UBP).^33^ Ligands were docked using Cresset Flare™ software. The docking score used was a Lead Finder (LF) dG score, which has been optimized for protein–ligand binding energy, Δ*G*, on the assumption that the pose of the compound is correct. A more negative score predicts a better binding. Initially, to check the docking efficacy, urea and AHA were docked. This confirmed whether the docking was performing as expected and allowed comparison to the literature (Fig. S1†). Compounds were designed based on known inhibitors, then the docking score and contacts with the protein were assessed and used to design further compound series (Fig. 1 and 8, Table S1†). Care was taken with the interpretation of the docking score.

**Fig. 2:**
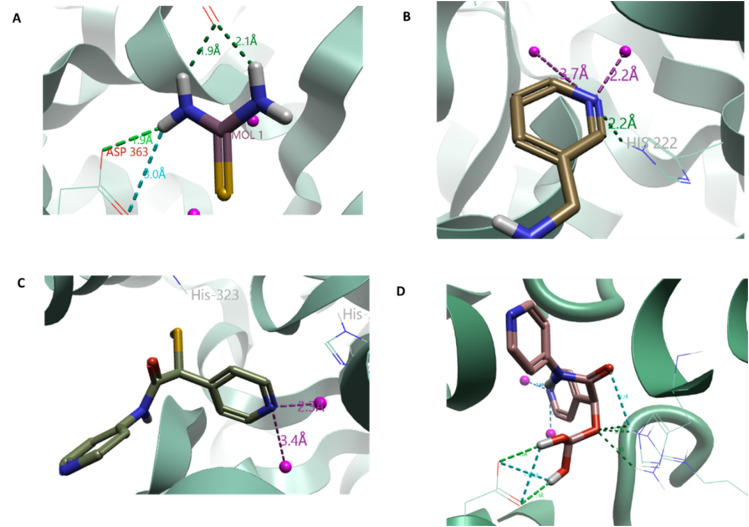
A. Thiourea docked into the active site of urease, distance measured between the amine hydrogen and D398 and G280, indicates hydrogen bond formation. B. A11 docked into the active site, showing the hydrogen bond formation between the pyridine ring and the H222 as well as the interactions with the Ni^2+^ ions. C. Results from the 2-mercaptoacetamide (2-MA) series (B); docking of B17 (R) (C2 (R)) show that the pyridine ring improved docking scores as seen in series A. D. Docking of C24 (S) indicating the interactions between the surrounding amino acids: D224, R339, and H323. Ni^2+^ ions shown as pink spheres. Urease, PDB 4UBP, shown as green ribbon, selected amino acids as thin sticks and compound docked as thick sticks. Molecules docked with Cresset Flare v. 4.0.2. Images generated using Flare™ from Cresset®.

Compounds with much higher molecular weights appear to have bias and therefore predict higher docking scores.^34^ Initial docking of the known inhibitor, thiourea, identified the formation of hydrogen bonds between the amine hydrogen on the ligand and Asp-383 and Gly-280, Fig. 2A (Fig. S2†). Therefore, in the design of series A, an amine hydrogen was kept, maintaining its H-bonding capacity. When compounds A1–A17 were docked, a carboxylic derivative appeared to hydrogen bond with His-222 and aligned the compound between the two Ni^2+^ ions. From the literature, a carboxyl group had been previously identified as coordinating with the Ni center, leading to increased urease inhibition.^28^ The pyridine ring also appeared to form hydrogen bond interactions between the His-222 and cation–pi interactions with N of Arg-339 (Fig. 2B). Series B was based on 2-mercaptoacetamide (2-MA), which has been shown to increase catheter lifetime in *in vitro* bladder models of a catheterized urinary tract.^20^ Furthermore, the pyridine ring from series A was also incorporated into the compounds, as it was found to increase the docking score. Surprisingly, B17 had the most negative score, with two pyridine rings (Fig. 2C). Series C was an optimization of series B. It was hypothesized that an increase in the length of the compound would improve hydrophobic interactions within the active site. Additionally, it was noted that the sulphide did not appear to be involved in contacts, so was replaced with a carbonyl. It was predicted that an oxygen would be less toxic than a sulphur and lead to fewer unwanted interactions^35^ (C21–24, Table S1†). The substitution of the sulphide with a carbonyl did not significantly affect the docking score, (C10 LF dG = −10.195 kcal mol^−1^*vs.* C24 (S) LF dG = −8.526 kcal mol^−1^), despite more contacts being identified visually, hence emphasizing the importance of visual assessments as well as analysis of the docking score (Fig. 2D) (Table S1†).

**Fig. 3:**
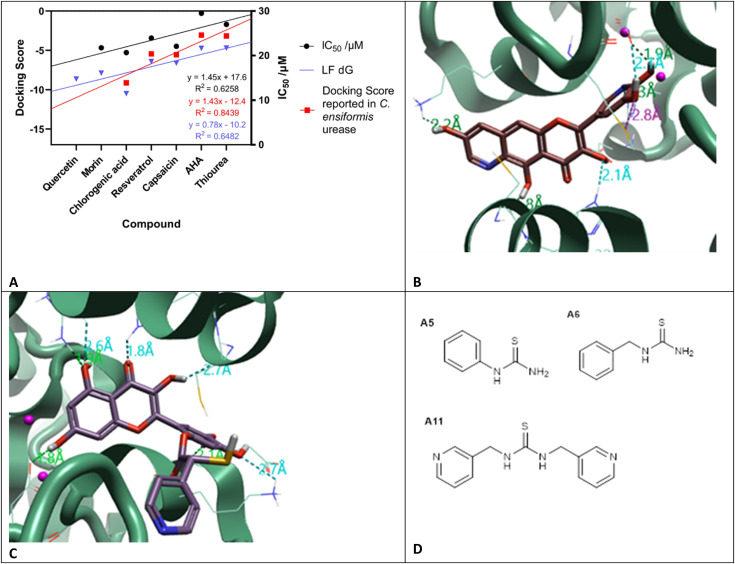
A. LF dG (blue) is the calculated docking score based on the docking results for the flavonoids, acetohydroxamic acid (AHA), and thiourea; docking score taken from Katrina *et al.*^37^ (red) calculated against urease from *Canavalia ensiformis* (PDB: 3LA4); *in vitro* IC_50_ taken from Xiao *et al.*,^36^ (black). Urease (PDB: 4UBP) was used for docking experiments. Molecules docked with Cresset Flare™ v. 4.0.2. B. Compound Diii2 docked into the active site of urease, with interactions with the active site flap, C322 and H323; as well as into the active site: H222, D363, and M367. C. Compound E5 (R) docked to urease, contacts made with the active site: D363, and R339; and the active site flap: C322 and H323. Urease (PDB: 4UBP) shown as green ribbon, selected amino acids as thin sticks and compound docked as thick sticks. Molecules docked and images created using Cresset Flare v. 4.0.2. D. Lead thiourea based compounds A5 (*N*-phenylthiourea), A6 (benzylthiourea) and A11*N*,*N*′-bis(3-pyridinylmethyl)thiourea (Bis-TU).

Flavonoids have been identified as potential urease inhibitors.^36^ To allow comparison to *in vitro* literature data, five flavonoids were docked and their docking score compared to reported *in vitro* IC_50_ data (concentration of the compound which reduces the activity of urease by half) and docking experiments using *Canavalia ensiformis* urease.^37^ A more negative docking score correlated with a lower IC_50_ and agreed with the trend of the docking score from *C. ensiformis* experiments reported by Kataria and Khatkar (Fig. 3A).^37^ This supported the docking methodology. Chlorogenic acid appears to have greater potency, compared to the other flavonoids; the extra length was thought to improve potency, owing to the greater likelihood of more contacts in the active site being made. Therefore, for series D an extra ring was added to quercetin to increase interactions. Compound Diii2 had the most negative docking score of −11.171 kcal mol^−1^; it had interactions with residues within the active site and outwards towards the edge of the protein (Fig. 3B). This supported the hypothesis that flavonoids bind more favorably to the active site flap, rather than directly into the active site center, as described for quercetin by Xiao *et al.* (Fig. S2†).^33,36^

Analysis of docking scores from series C and D led to the conclusion that structures based on 2-MA could be used as a warhead, directly interacting within the active site and binding to residues involved in the catalytic mechanism, whilst flavonoid-based compounds interact with the active site flap. Series E incorporated the warhead, 2-MA, with the flavonoid scaffold. Compound E5 (S) had the most negative docking score (−12.902 kcal mol^−1^), and made interactions with Cys-322, known to be present in the mobile active site flap (Fig. 3C).^38^ The docking of these larger ligands was slightly limited by the size of the grid box; if these compounds are further investigated as inhibitors a larger grid box would be required for *in silico* experiments.

Filtering the *in silico* screen: identifying structure–activity relationships (SAR) and determining commercial availability. As described in Fig. 8, the compounds were then screened using Lipinski's rule of 5.^39^ The empirical ‘rule of 5’ states that drug-like compounds should have <5 hydrogen bond donors (HBD), <10 hydrogen bond acceptors (HBA), molecular weight <500 Da and log *P* <5. These are good parameters for oral drug delivery, but also apply for local delivery through the Biomodics IPN catheters; as delivery through a membrane depends on permeability, which is a product of diffusivity and solubility.^40^ Lipinski's rule of 5 provided a filter for the results of the *in silico* screen. SAR were identified; all top 20 compounds contained a carbonyl group, top compounds from series C, D, and E all contained a catechol moiety, and a pyridine ring was found in 18 of the top 20 compounds.

Hydrophobic domains were common in compounds with high docking scores, agreeing with previous studies showing hydrophobic behavior to be successful in urease inhibition.^28^ The top compounds identified were predominantly found in the flavonoid series (D), however assessment of the accessibility of these compounds, either by purchasing or synthesizing, highlighted that these compounds are difficult to produce. As this is the first run of what could be an iterative screening process, it was decided to focus on the thiourea series as these compounds could be purchased (Fluorochem, UK). The following compounds were purchased for *in vitro* testing: A5 (*N*-phenylthiourea), A6 (benzylthiourea), and A11 (*N*,*N*′-bis(3-pyridinylmethyl)thiourea (Bis-TU)).

### *In vitro* testing

**Fig. 4:**
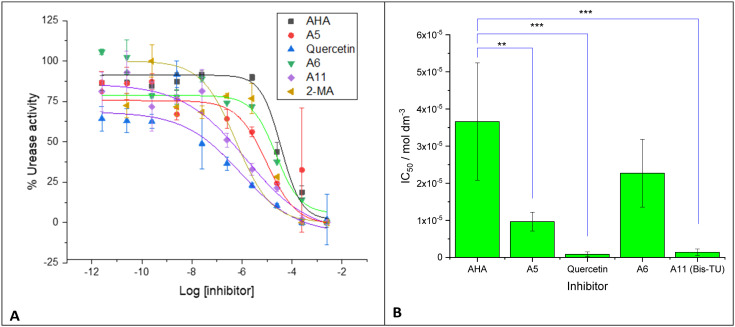
A. *Proteus mirabilis* urease activity measured with compounds: acetohydroxamic acid (AHA) (red), 2-mercaptoacetamide (2-MA) (green), quercetin (purple), A6 (benzylthiourea, brown), A5 (*N*-phenylthiourea, dark green), and A11 (*N*,*N*′-bis(3-pyridinylmethyl)thiourea (Bis-TU), blue). IC_50_ calculated by fitting response to a sigmoidal dose–response curve (see Table S3†). Experiments were completed with three biological repeats, two technical repeats, graphs show the mean of the repeats with error bars representing standard deviation. B. Plot of IC_50_ from fitting in A for tested compounds. Note, 2-MA did not fit to the sigmoidal dose–response curve. Graphs and fitting using Origin Pro. Error bars represent the standard deviation of four independent replicates, analyzed on GraphPad using an unpaired t test: *p* < 0.01 (**); *p* < 0.001 (***).

The three compounds: A5 (*N*-phenylthiourea), A6 (benzylthiourea) and A11*N*,*N*′-bis(3-pyridinylmethyl)thiourea (Bis-TU) were initially tested against urease-expressing whole cell *P. mirabilis* cultures. Urease in *P. mirabilis* is intracellular so this assay also tests the ability of the compound to cross the outer bacterial membrane and access the urease within the periplasm, moreover *P. mirabilis* is the principal pathogen implicated in urinary catheter blockage.^41^ IC_50_ was determined for each compound; known urease inhibitors were also run to compare against the newly identified compounds: AHA,^21^ 2-MA,^20^ and quercetin (Fig. 4 and Table 1), fitting results shown in Table S3.†^36^ Results from the whole cell *P. mirabilis* urease showed that both quercetin and A11 (Bis-TU) had a *ca.* 25-fold lower IC_50_ than AHA and again A5 and A6 outperformed the control compound AHA (Table 1), although A6 was not statistically significantly lower. As A11 (Bis-TU) had the lowest IC_50_ of the thiourea compounds, it was selected for further study. 2-MA did not fit to a sigmoidal dose–response curve, hence IC_50_ could not be estimated.

**Table 1:** IC_50_ determined from Fig. 4 for urease expressed in whole cell *Proteus mirabilis*. Uncertainty represents the standard deviation of three independent replicates, and *p* values analyzed on GraphPad using an unpaired *t* test

	*Proteus mirabilis*	*p vs.* AHA
	IC_50_/mol dm^−3^	
AHA	3.66 × 10^−5^ ± 1.58 × 10^−5^	
A5	9.66 × 10^−6^ ± 2.54 × 10^−6^	0.0021
Quercetin	8.66 × 10^−7^ ± 6.47 × 10^−7^	0.0002
A6	2.27 × 10^−5^ ± 9.13 × 10^−6^	NS (0.0916)
A11 (Bis-TU)	1.40 × 10^−6^ ± 8.86 × 10^−7^	0.0003
2-MA	No fit	

The urease inhibitors were designed to be anti-virulence compounds and are used to knock out the urease function and not antimicrobials. The ability of the compounds to inhibit bacteria was assessed using *P. mirabilis* and *E. coli*, common causes of CAUTI (Fig. S4, Table S2†).^42^ AHA and Bis-TU appeared to have only a modest inhibitory effect on *E. coli* and *P. mirabilis* growth. As almost all long-term catheter users have CAUTI (asymptomatic or symptomatic), the aim was to prevent catheter blockage rather than inhibit bacterial growth: thus reducing selection pressure for evolution of resistant bacteria.

### Drug delivery

Biomodics ApS have developed a drug-delivery urinary catheter, whereby the balloon of the catheter is made of an IPN. The IPN consists of silicone, the usual material for long-term catheters, modified with the hydrogel poly(HEMA-*co*-PEGMEA).^43^ The hydrogel imparts a degree of permeability into the catheter, providing a sustained drug delivery when incorporated into the IPN.^31^ Therefore, the delivery system prevents immediate exposure to potentially toxic concentrations of the drug. Critically, release of the API is sustained locally to the site of infection. In comparison to oral delivery, this enables a low dose to achieve the same therapeutic effect in addition to ensuring greater bioavailability for lower solubility formulations.

**Fig. 5:**
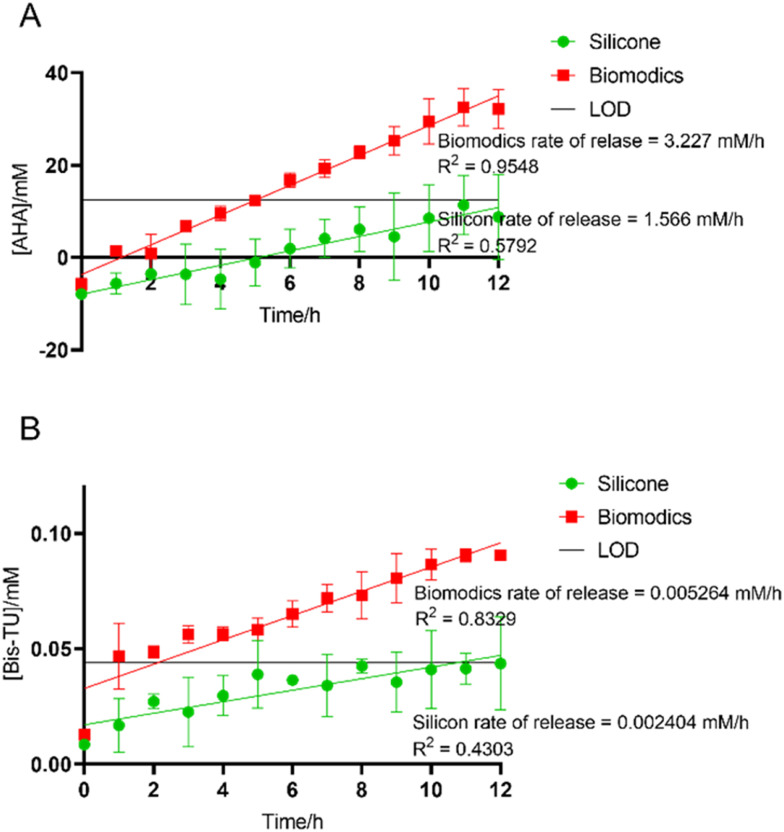
A. Release of acetohydroxamic acid (AHA). B. Release of *N*,*N*′-bis(3-pyridinylmethyl)thiourea (Bis-TU (A11)). Measured across the balloon of the Biomodics catheter and silicone catheter over 12 h. Experiments were completed with three repeats, graphs show the mean of the repeats with error bars representing standard deviation, simple linear regression analysis has been completed to get a line of best fit and limit of detection (LOD) is shown. Graphs produced using GraphPad Prism v. 9.4.1.

Additionally, local delivery can reduce unwanted side effects; AHA is toxic when delivered systemically. To demonstrate that AHA and A11 (Bis-TU) had the capability to diffuse across the balloon and be delivered into the bladder, UV-vis spectroscopy experiments were used to detect the delivery of the drugs across the balloon in artificial urine (Fig. 5). The release rate from the Biomodics IPN catheter was compared to a standard silicone catheter used by long-term catheter users. For both compounds there was no release from the standard silicone catheters above that of the limit of detection (LoD), whilst the Biomodics catheters demonstrate zero order kinetics release over the 12 h experimental period. Therefore, Bis-TU and AHA can be delivered through the Biomodics IPN catheter.

### *In vitro* bladder models

**Fig. 6:**
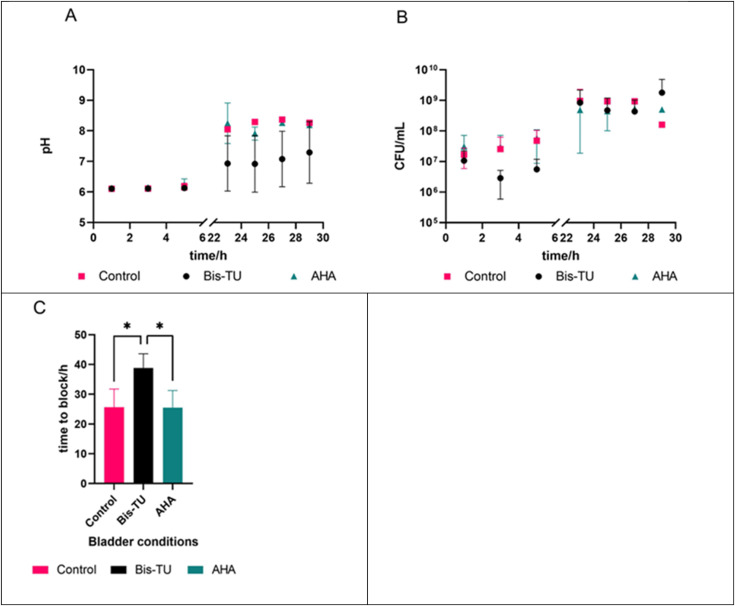
A. Monitoring of the *in vitro* bladder models' pH over time, comparing bladders treated with: acetohydroxamic acid (AHA), *N*,*N*′-bis(3-pyridinylmethyl)thiourea (Bis-TU) and no treatment (control). B. Monitoring the CFU mL^−1^ of the models over time. C. Comparing the blockage time between each of the bladder models. Experiments were completed with three repeats, graphs show the mean of the repeats with error bars representing standard deviation, * indicates a *p* = 0.0426 calculated by unpaired *t*-test. Graphs and calculations produced using GraphPad Prism v. 9.4.1.

The *in vitro* bladder model of a catheterized urinary tract enables the simulation of the crystalline biofilm and measurement of the blockage of the catheter caused by a *P. mirabilis* infection.^44,45^ The artificial bladders were infected with a high inoculum of bacteria to mimic a late-stage CAUTI, average inoculation was 3.36 × 10^7^ CFU mL^−1^. Each model was catheterized with a Biomodics IPN catheter, and the balloons were inflated with 20 mM AHA in a 9 : 1 mixture of saline and DMSO; 20 mM Bis-TU in a 9 : 1 mixture of saline and DMSO; and the control bladder a 9 : 1 mixture of saline and DMSO. The artificial bladders were allowed to equilibrate over 18 h before inoculation. There is approximately a four-fold dilution during the diffusion of the compound in the artificial urine (∼5 mM at the start), however, this concentration changes once the pumps start because of the continual dilution from the filling of the bladder from the ‘kidneys’ and the drainage *via* the catheter. Monitoring of the pH and CFU mL^−1^ in the bladder allowed tracking of the experiment (Fig. 6A and B). Bis-TU was able to keep the pH lower than that of the AHA and control bladders (Fig. 6A), whilst the quantity of bacteria were comparable across all bladders (Fig. 6B). The blockage of the catheters was the end of the experiment and allowed determination of whether the compounds could increase the lifetime of the catheter (Fig. 6C). Bis-TU significantly outperformed the clinical standard AHA, and the control, indicating that Bis-TU has anti-ureolytic activity (unpaired *t*-test, GraphPad Prism 9.4.1, *p* = 0.0366 and *p* = 0.426 respectively).

### Cytotoxicity analysis

**Fig. 7:**
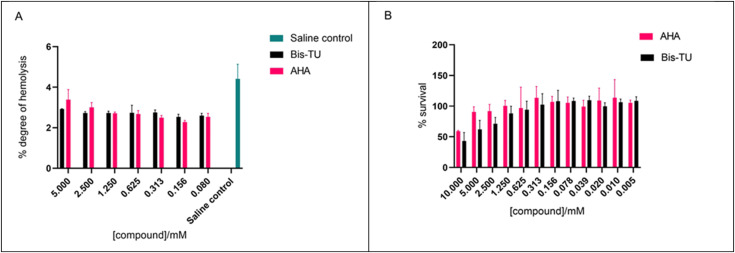
A. *Ex vivo* haemolytic assay to test the toxicity of acetohydroxamic acid (AHA) and *N*,*N*′-bis(3-pyridinylmethyl)thiourea (Bis-TU). B. HepG2 mammalian cell cytotoxicity experiment with AHA and Bis-TU assessed over 24 h. Experiments were completed with three biological repeats, graphs show the mean of the repeats with error bars representing standard deviation. Graphs and calculations produced using GraphPad Prism v. 9.4.1.

The ability of AHA and Bis-TU to cause haemolysis was assessed using an *ex vivo* haemolytic assay. Results indicate that Bis-TU and AHA do not cause haemolytic activity (Fig. 7A). The methyl thiazolyl tetrazolium (MTT) assay was used to test the effect of the compounds on HepG2 cells; these liver cells are often used to evaluate toxicity during pharmacological research.^46^ At high concentrations, 10 mM, both Bis-TU and AHA affect the survival of HepG2 cells within the 24 h of incubation. As the concentration decreases AHA appears less cytotoxic, however at concentrations below 1.25 mM there is no difference between the effect of AHA and Bis-TU (Fig. 7B). Although the concentrations used in the *in vitro* bladder model experiment where higher than 1.25 mM (∼5 mM) the Biomodics IPN catheter delivery mechanism means that the effective concentration in the bladder is below the toxic concentration and hence the effect of the toxicity is reduced.

## Conclusions

Bis-TU is a newly identified urease inhibitor, which significantly extends the lifetime of a urinary catheter compared to AHA. It was discovered by a rationally designed *in silico* screen coupled with rigorous *in vitro* methods, thus enabling a cost-effective drug discovery process. This methodology is underpinned by the following: (1) strong previous literature to design the screen,^28,29^ (2) a high-resolution crystal structure, and (3) a physiologically representative *in vitro* model. This approach could be used to target other virulence factors to treat further diseases. Overall, we believe the targeting of bacterial virulence factors, rather than the bacteria themselves may be a pragmatic approach to development of new drugs for infection control, since it could reduce selection pressure on bacteria to evolve resistance, in contrast to antibiotic drugs.

Additionally, the screening method can be repeated in an iterative manner to improve the outcome. For example, the learning from the first screen could be fed back into another round, therefore improving the potency of Bis-TU, and potentially incorporating the learning from the flavonoid screen to produce compounds which can easily be synthesized. The limitation of Bis-TU is its relatively low solubility: future work could involve the addition of an excipient which could improve solubility and could be incorporated into the balloon inflation solution to allow more effective delivery at higher concentrations. Here, we have also shown a drug delivery technique which allows directed local delivery of Bis-TU into the bladder. It should be noted that the data shows that quercetin, found in plants including watercress, has an IC_50_ comparable to Bis-TU, which suggests that it may have utility as a nature derived urease inhibitor suggesting the utility of a parallel drug development approach.

## Experimental

### Computational docking methodology

**Fig. 8:**
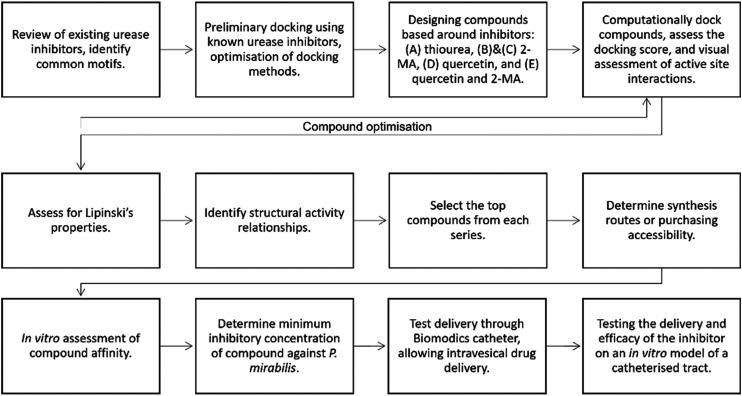
Flow diagram depicting the strategy for identifying a new urease inhibitor.

A summary of the inhibitor discovery process is shown in Fig. 8. The urease crystal structure from *Sporosarcina pasteurii* was used to carry out the *in silico* docking experiments; this had a high resolution of 1.55 Å (PDB code: 4UBP).^33^ Although this is not the crystal structure from the *P. mirabilis* urease, it has a conserved active site and has been used previously for modelling compound docking.^20^ Cresset Flare™ 4.0.2 (revision: 40719, Cresset, Litlington, Cambridgeshire, UK) software was used to prepare the crystal structure and complete the docking experiments.^47–49^ The ‘accurate but slow’ docking setting was used by the Flare software. The compound structures were prepared in ChemDraw 19.1.1.21 (PerkinElmer Informatics, Waltham, Massachusetts, US).

A 10 Å grid box was prepared around Ni 6057 (C Ni 798) found in the active site of the protein. The crystal structure contains crystallized AHA which allowed for control docking experiments. AHA was computationally docked onto the protein, the docked coordinates of the compound were compared to the crystallized coordinates of AHA, and the root mean squared deviation (RMSD) was used to compare the coordinates. The RMSD was 0.997 Å, which indicates the AHA is well docked, as any compound with a RMSD <2 Å is considered to be performing well and would accurately predict the docking of further compounds.^50^ Known urease inhibitor 2-MA and the substrate, urea, were docked into the active site, allowing optimization of the method.

### Designing the compound series

A series of compounds were designed with inspiration from known urease inhibitors; (A) thiourea,^24,29,51,52^ (B) & (C) 2-MA,^20^ (D) quercetin,^36,53^ and (E) quercetin and 2-MA.^20^ Compounds were assessed for their HBD and HBA groups, hydrophobic chains, aromatic groups, and sulphur-containing groups. Any compound containing chiral centers was docked in all configurations. Structures of all compounds can be found in ESI Fig. S3;† this has been summarized in Fig. 1. Contacts to the active site were assessed, and number of contacts <3.5 Å were counted. Compounds were optimized with additional changes to the R-groups depending on docking score and docking pose in the active site (Fig. 1). The design of the compounds was not limited by synthetic or purchasing accessibility, such that the design of the compounds was not constrained.

### *In vitro* assays

#### Urease activity assay

The *in vitro* assay used to measure the urease activity was the Berthelot assay, which measure the accumulation of ammonia.^54,55^ Compounds were tested using whole-cell *P. mirabilis* B4 (from the Jenkins Group collection, University of Bath). *P. mirabilis* was cultured overnight, 37 °C with 200 rpm shaking in 10 mL of Luria-Bertani broth (LB, Merck, Germany). The overnight culture was centrifuged, 3100 g, 10 min, at 4 °C (5810 R Eppendorf); the supernatant was discarded, and the pellet resuspended in phosphate buffer saline (PBS, Fisher Scientific, UK). In each well of a 96-well plate (Corning) 10 μL of 0.5% sulfuric acid was added. *P. mirabilis* (100 μL, 0.004 mg mL^−1^) in phosphate buffer was incubated with the desired compound concentration and urea (100 μL, 50 mM in phosphate buffer) for 30 min at 37 °C.

Experiments were run in triplicate of two technical replicates (*n* = 6), with 10-fold dilutions of the compounds including a positive and negative control. To each well, 20 μL of 60 mM sodium hydroxide is added. Solution A (50 μL, 106 mM phenol, 191 μM sodium nitroprusside) and solution B (50 μL, 125 mM sodium hydroxide, 125 mM sodium hypochlorite) were added to each well and the plate was incubated for 30 min at 37 °C. The absorbance of the wells was read at 636 nm (SPECTROstar Omega BMG LabTech, Germany). Urease activity was calculated using eqn (1). IC_50_ was determined using non-linear regression (GraphPad Prism v9).1
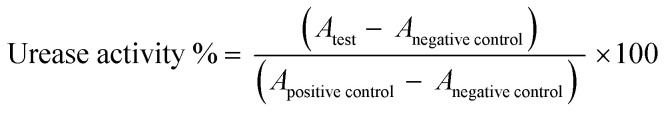


#### Minimum inhibitory concentration (MIC) assay

The compound was dissolved in sterile water with 5% (v/v) DMSO. To the first column on a 96-well plate (Corning, UK), 200 μL of compound was added, it was then serially diluted two-fold (100 μL) across the plate to column 10. A subculture of *P. mirabilis* (1 × 10^6^ CFU mL^−1^) or *E. coli* NSM59 (1 × 10^6^ CFU mL^−1^, from the Jenkins Group collection, University of Bath), 100 μL, was added to all columns 1–10. LB broth only was added to column 11, the negative control and 200 μL of *P. mirabilis*/*E. coli* subculture was added to column 12, the positive control. The plate was incubated at 37 °C for 18 h, using a SPECTROstar Omega plate reader (BMJ LabTech) the absorbance at 600 nm was measured at regular time points during the 18 h incubation. The plate was shaken for 10 s, at 200 rpm prior to a reading being taken. The MIC was defined as the lowest concentration of compound that inhibited growth and the highest concentration that permitted bacterial growth. Initially, this was done through visualization of the plate after 18 h but also through examination of the growth curves measured at OD_600_.

#### Quantifying delivery through the Biomodics catheter

Delivery through the permeable balloon was measured using UV-vis spectroscopy with a quartz cuvette (pathlength 1 cm) (Agilent Cary 60 UV-vis spectrometer). Calibration curves for AHA (Sigma) and *N*,*N*′-bis(3-pyridinylmethyl)thiourea (Flurochem, Bis-TU (A11)) were prepared using absorbances 235 nm and 239 nm, respectively (Fig. S5†). Sterile 50 mL beakers were used to mimic the bladder; catheters were inflated with compound solutions, AHA (320 mM) or Bis-TU (85 mM). Biomodics IPN catheters were compared to standard all-silicone catheters (Tiga-Med, Germany). Beakers were filled with artificial urine, prepared according to Nzakizwanayo *et al.*, 30 mL was added to the beakers which were sealed with parafilm and incubated at 37 °C.^45^ Every hour for 12 h, 1 mL was removed from the artificial urine surrounding the catheter balloon and the absorbance spectra was measured. The 1 mL was returned to the beaker, so the volume remained constant. The quantity of compound released over time was determined using the calibration curves. Limit of detection (LOD) was calculated by eqn (2), where *σ* is the standard deviation of the lowest concentration.2
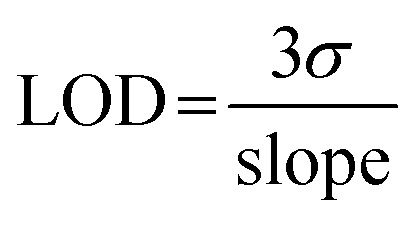


#### *In vitro* model of the catheterized urinary tract

*In vitro* bladder models were set up as described by Nzakizwanayo *et al.*, the flow rate was set to run at 0.8 mL min^−1^ using a Watson-Marlow 323S/D (030.3134.3DU) pump.^45^ The artificial bladders were maintained at 37 °C and catheterized with Biomodics IPN catheters (donated by Biomodics ApS). Balloons were inflated with 10 mL of the following solutions: control bladder: a 9 : 1 mixture of saline (150 mM sodium chloride) and DMSO; Bis-TU bladder: 20 mM Bis-TU in a 9 : 1 mixture of saline and DMSO; and AHA bladder: 20 mM AHA in a 9 : 1 mixture of saline and DMSO. The models were set up and filled with artificial urine until they started to drain, the pump was then paused, and the compound solution allowed to equilibrate with the artificial urine in the bladder for 18 h. *P. mirabilis* B4 (from the Jenkins Group bacteria collection) was grown at 37 °C overnight with 200 rpm shaking in 10 mL of LB (Merck, Germany). After 18 h of growth the culture was centrifuged at 3100*g*, 10 min, 4 °C (5810 R Eppendorf); the supernatant was discarded, and the pellet resuspended in PBS (Fisher Scientific, UK). Resuspended *P. mirabilis* was diluted ×100 with PBS, volume of 5 mL, and a sample was taken. An average of 3.7 × 10^7^ CFU mL^−1^ of *P. mirabilis* was added to the artificial bladder, to mimic a late-stage CAUTI. Inoculation and CFU was determined by the Miles and Misra quantification technique.^56^ At regular time intervals during the experiment, a sample was taken aseptically from each artificial bladder for bacterial quantification and to measure the pH. The end of the experiment was determined when the bladders blocked. Additionally, the artificial bladders were monitored by time-lapse photography overnight using a Nikon D3100 camera with photos taken every 2 min.

### Cytotoxicity testing

#### Ethics

For the haemolysis assay, three volunteers gave written consent prior to donation of whole blood by a trained phlebotomist at the University of Bath, which was pooled. Recruitment commenced on 3 February 2023 and ended on 7 February 2023. This project was approved by the University of Bath, Research Ethics Approval Committee for Health (REACH) [reference: EP 18/19 108].

#### *Ex vivo* haemolysis assay

Initial solutions of AHA and Bis-TU were prepared at a concentration of 10 mM with 1% DMSO in PBS. Varying concentrations were prepared by serial dilution across a 96-well plate (100 μL). Whole blood was obtained from three consenting donors and drawn directly into lithium heparin-coated vacutainer tubes. The whole blood was centrifuged at 500*g* for 10 min at 4 °C (5810 R Eppendorf), supernatant (plasma) was removed and replaced with double the original volume of saline solution (150 mM sodium chloride (Merck, Germany)). The erythrocyte pellet was re-suspended and re-centrifuged. The erythrocytes were washed three times. The erythrocyte pellet was diluted 1% (v/v) with saline, then incubated in a 96-well plate (100 μL) with varying concentrations of AHA and Bis-TU (5–0.08 mM), saline (negative control) and 1% triton (positive control) (100 μL); for 1 h at 37 °C under steady rotation. The plate was then centrifuged at 500*g* for 5 min and the supernatant transferred to a new 96-well plate. The absorbance was measured at 404 nm (Tecan Sunrise) and the degree of haemolysis was calculated according to eqn (3).3
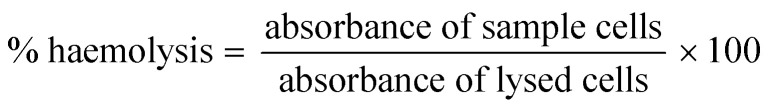


#### HepG2 mammalian cell viability

Freezer stocks of HepG2 cells were defrosted and re-suspended in Dulbecco's Eagle medium (DMEM) complete (Gibco, this contains minimum essential media (MEM, Gibco)). A T75 flask (Nunc) was seeded with 10 mL of complete DMEM. Cells were grown for 3–4 days, a 37 °C, 5% CO_2_, to achieve adherent cells. Old media was removed and 5 mL of PBS added to wash the cells, this was then aspirated. Cells were incubated with 3 mL, 0.25% trypsin (Gibco), and PBS (previously warmed to 37 °C) for 7 min at 37 °C. After incubation cells were checked using a microscope (Nikon TMS inverted phase contrast) to confirm that they were no longer adherent. Cells were dissociated with 4 mL of media and re-suspended by pipetting 10 mL approximately 20 times. Cells were centrifuged for 3 min at 1000 rpm, media was aspirated, and pellet was resuspended in 1 mL of media. Cells were counted using a hemocytometer and new flasks were reseeded with 200 μL of cells to 10 mL of media. To maintain stocks, cells were sub-cultured every 2–3 days.

Compounds, AHA and Bis-TU, were prepared at 10 mM with 1% DMSO in DMEM media. Varying concentrations of compounds were prepared by serial dilution across at 96-well plate (100 μL). MTT (Invitrogen) assay was used to measure the metabolic activity of the HepG2 cells. MTT (1 mg mL^−1^) in media was filter sterilized and cell culture plates, 96-well (Nunc), were seeded with 1 × 10^4^ cells per well and grown for 24 h. Media/compound was removed and MTT (100 μL) was added for 60 min at 37 °C and then removed. Isopropanol (150 μL) was added and the plate was incubated in foil for 15 min on an orbital shaker. Absorbance was measured at 590 nm (reference filter at 620 nm) (Sunrise, Tecan). The % survival was calculated as per eqn (4).4
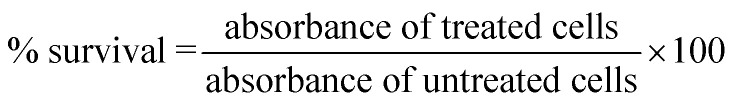


## Ethics statement

All experiments involving human tissue were performed in accordance with the Declaration of Helsinki and Guidelines of the University of Bath Research Ethics Approval Committee for Health reference number: EP 18/19 108, and experiments were approved by this ethics committee at the University of Bath. Informed consents were obtained from human participants of this study, who were over 18 years of old and able to consent.

## Data availability

Data provided in the ESI† and on request to the corresponding author.

## Conflicts of interest

There is no conflict of interest to declare.

## Supplementary Material

MD-OLF-D4MD00378K-s001
