# Supplementary material for: Rational design and in vitro testing of new urease inhibitors to prevent urinary catheter blockage
Source: RSC Med Chem. 2024 Sep 12;15(10):3597–608. doi: 10.1039/d4md00378k (PMC11391341; doi:10.1039/d4md00378k)
Supplement: MD-015-D4MD00378K-s001 [file MD-015-D4MD00378K-s001.pdf]

Supplementary Information (SI) for RSC Medicinal Chemistry.
This journal is © The Royal Society of Chemistry 2024

Supplementary data: Rational design and in-vitro testing of new urease inhibitors to prevent urinary catheter blockage, Heylen et al.

Supplementary Information

Series A

A1

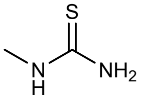

A2

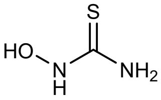

A3

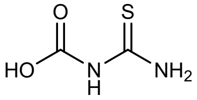

A4

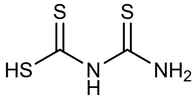

A5

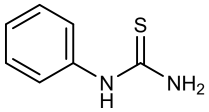

A6

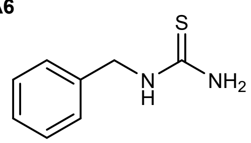

A7

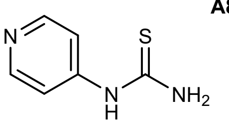

A8

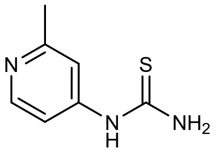

A9

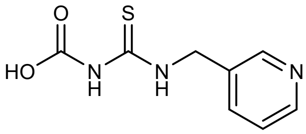

A10

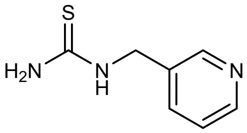

A11

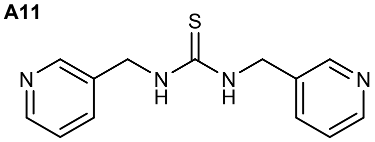

A12

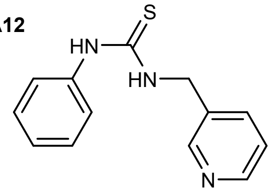

A13

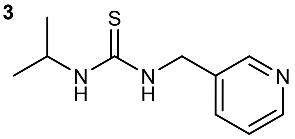

A14

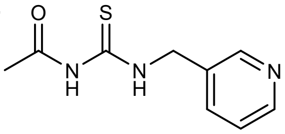

A15

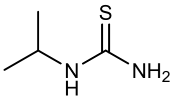

A16

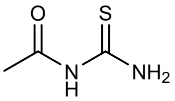

Supplementary data: Rational design and in-vitro testing of new urease inhibitors to prevent urinary catheter blockage, Heylen et al.

Series B

B1

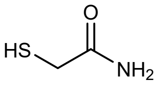

B2

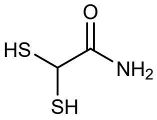

B3 (R)

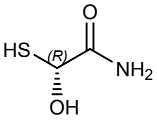

B3 (S)

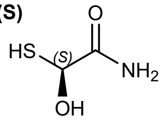

B4

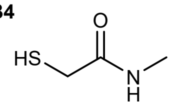

B5

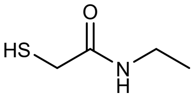

B6 (R)

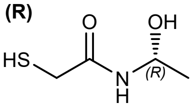

B6 (S)

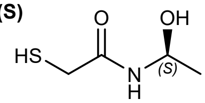

B7 (R,R)

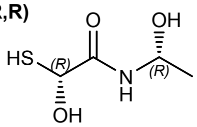

B7 (R,S)

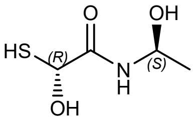

B7 (S,R)

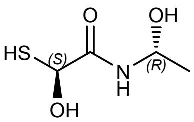

B7 (S,S)

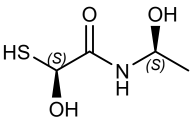

B8 (R)

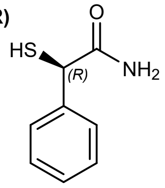

B8 (S)

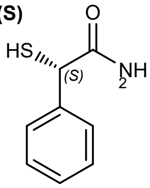

B9 (R)

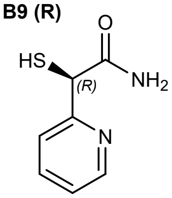

B9 (S)

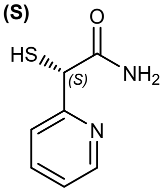

B10 (R)

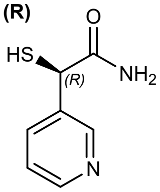

B10 (S)

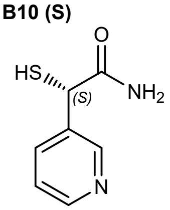

B11 (R)

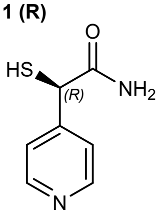

B11 (S)

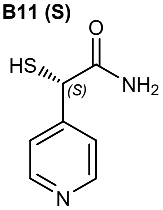

B12

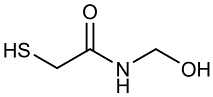

B13

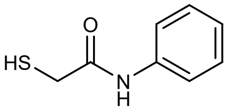

B14

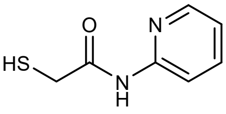

B15

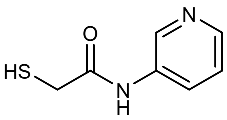

B16

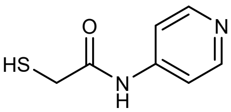

B17 (R)

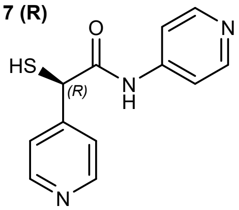

B17 (S)

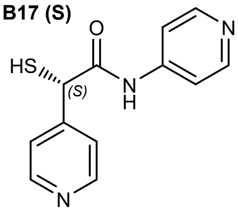

B18

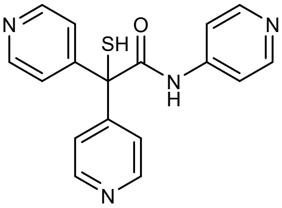

Supplementary data: Rational design and in-vitro testing of new urease inhibitors to prevent urinary catheter blockage, Heylen et al.

Series C

C1 (R)

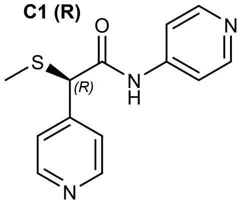

C1 (S)

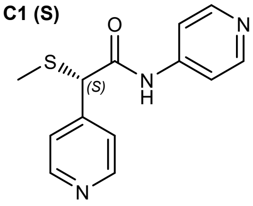

C2 (R)

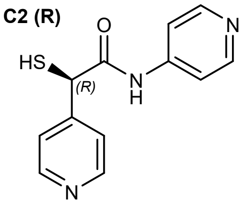

C2 (S)

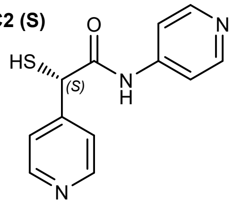

C3 (R)

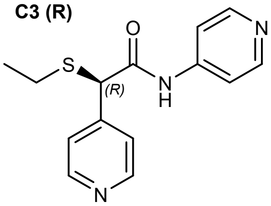

C3 (S)

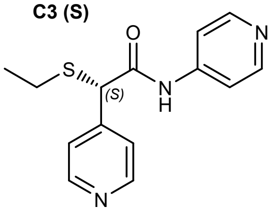

C4 (R)

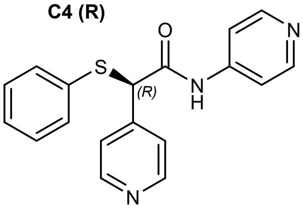

C4 (S)

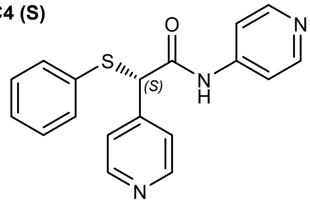

C5 (R)

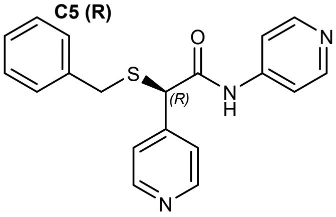

C5 (S)

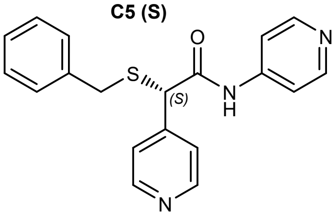

C6 (R)

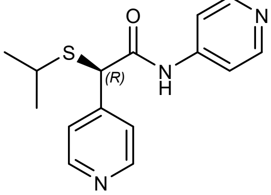

C6 (S)

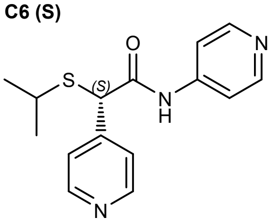

C7 (R)

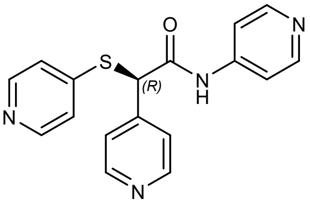

C7 (S)

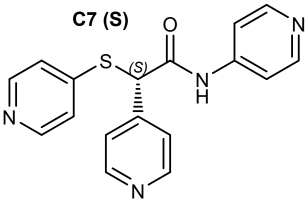

C8 (R)

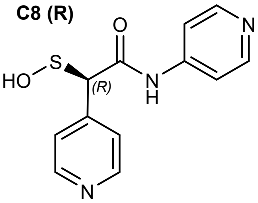

C8 (S)

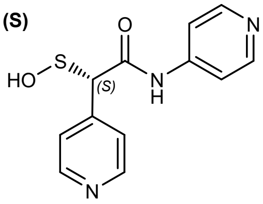

C9 (R)

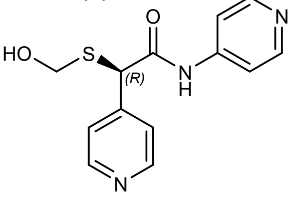

C9 (S)

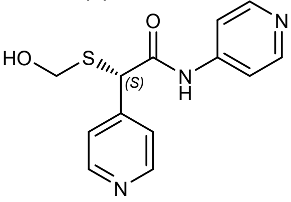

C10 (R)

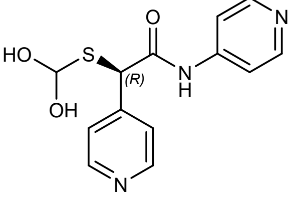

C10 (S)

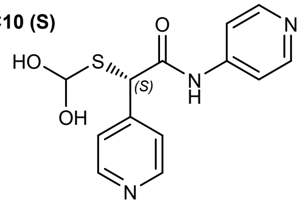

C11

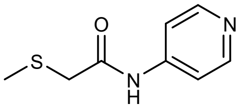

C12

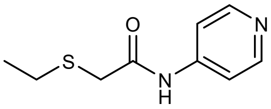

Supplementary data: Rational design and in-vitro testing of new urease inhibitors to prevent urinary catheter blockage, Heylen et al.

C10 (S)

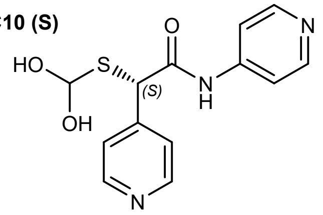

C11

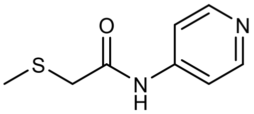

C12

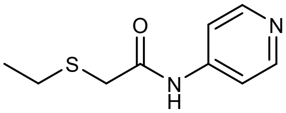

C13

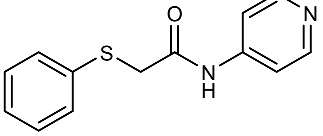

C14

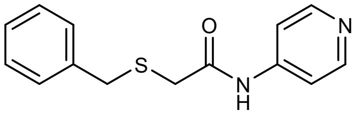

C15

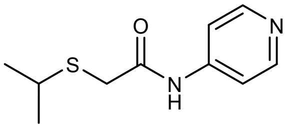

C16

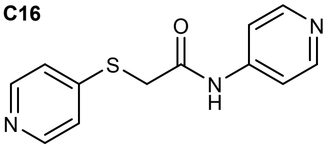

C17

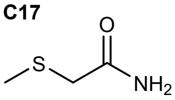

C18

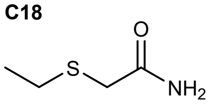

C19

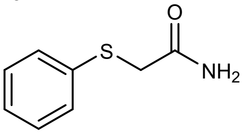

C20

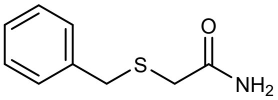

C21

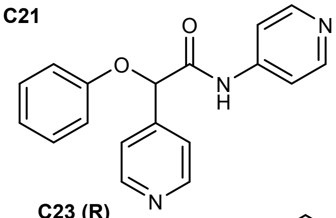

C22 (R)

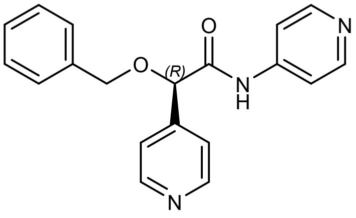

C22 (S)

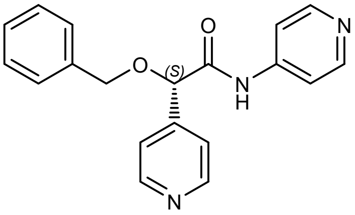

C23 (R)

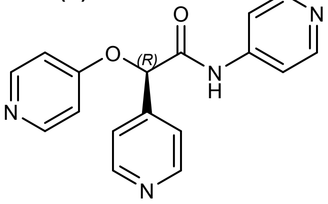

C23 (S)

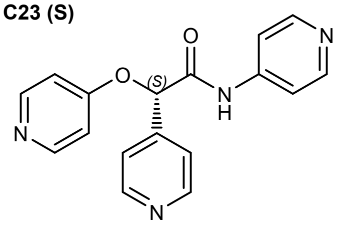

C24 (R)

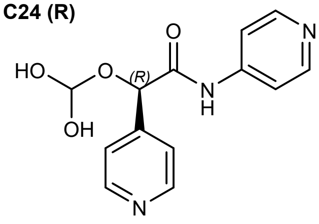

C24 (S)

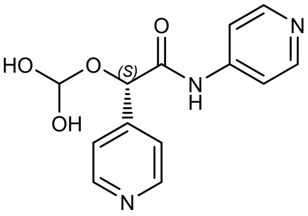

Supplementary data: Rational design and in-vitro testing of new urease inhibitors to prevent urinary catheter blockage, Heylen et al.

Series D

Di1

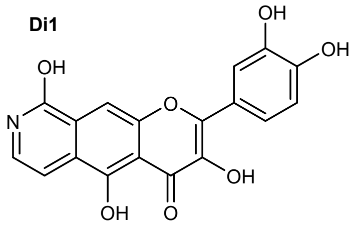

Di2

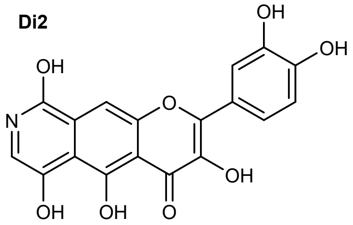

Dii1

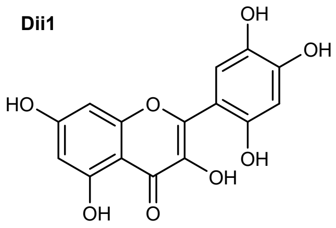

Dii2

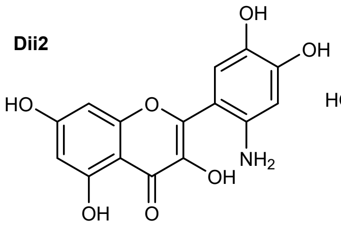

Diii1

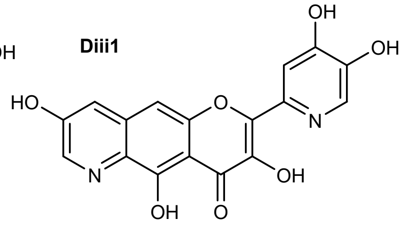

Diii2

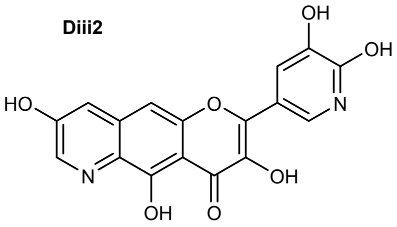

Diii3

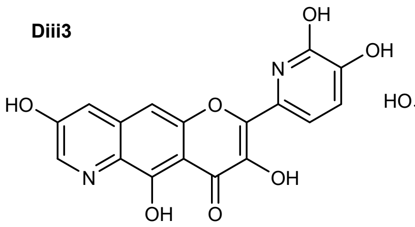

Diii4

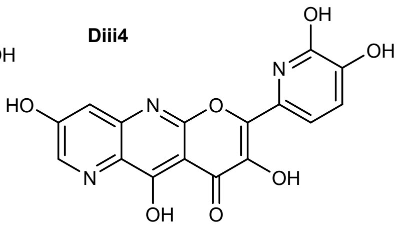

Div1

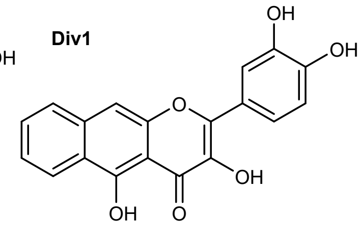

Div2

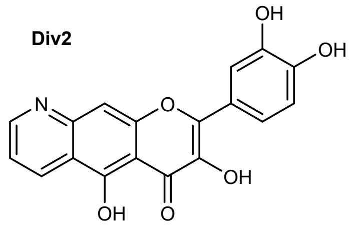

Div3

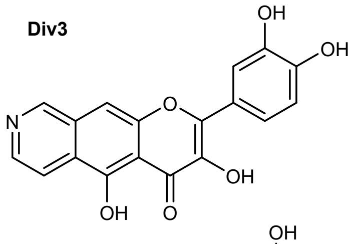

Div4

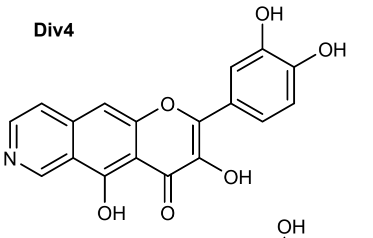

Div5

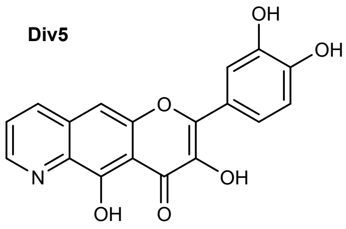

Div6

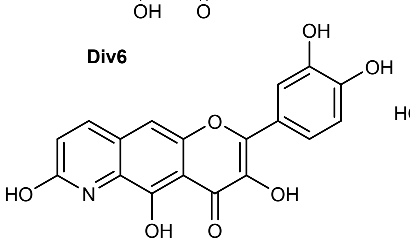

Div7

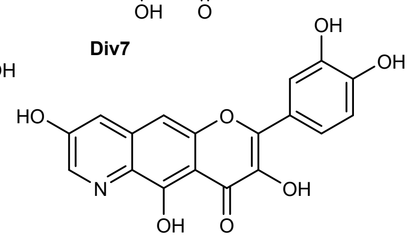

Div8

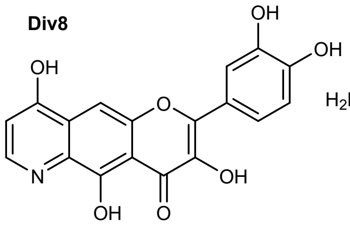

Div9

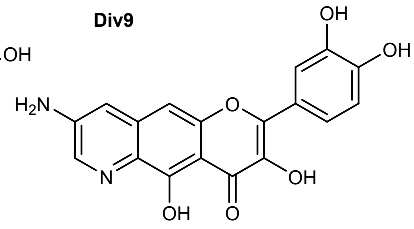

Dv1

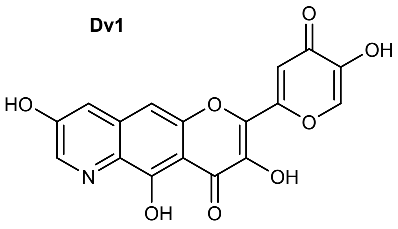

Dv2

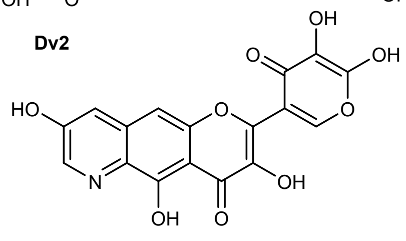

Dv3

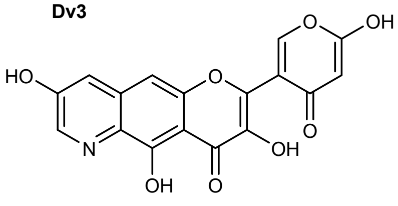

Supplementary data: Rational design and in-vitro testing of new urease inhibitors to prevent urinary catheter blockage, Heylen et al.

Series E

E1

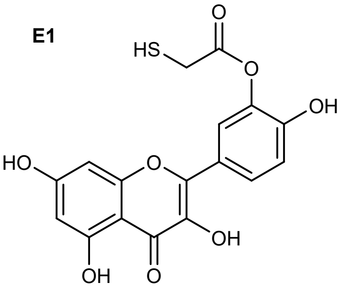

E2

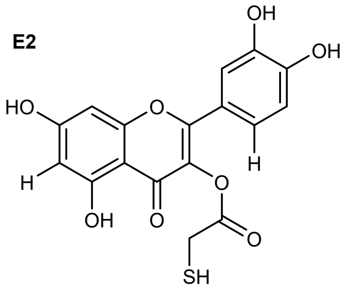

E3

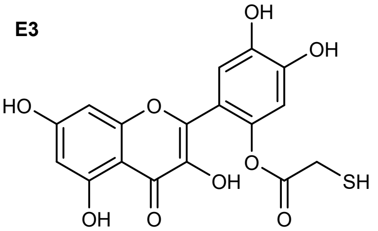

E4

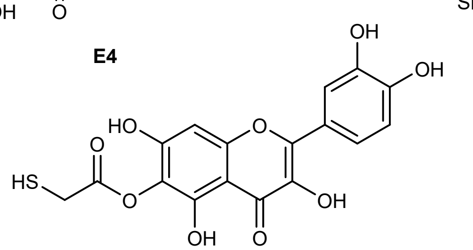

E5 (R)

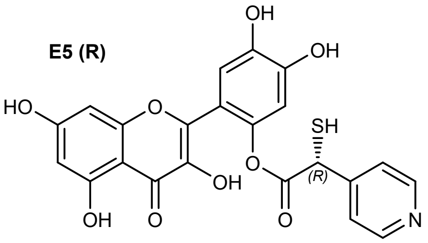

E5 (S)

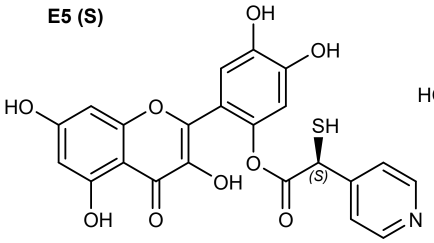

E6

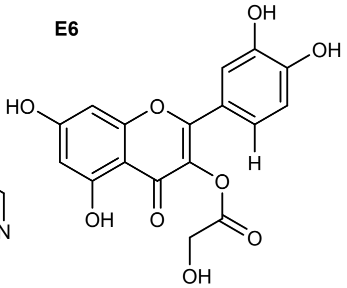

E7

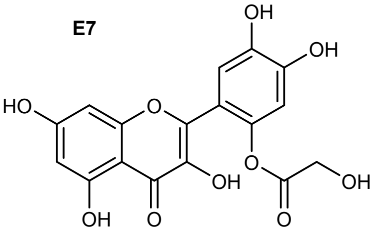

E8

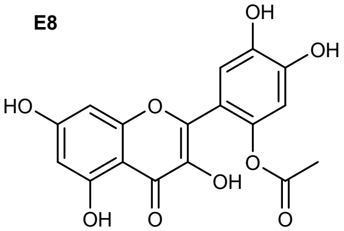

E9

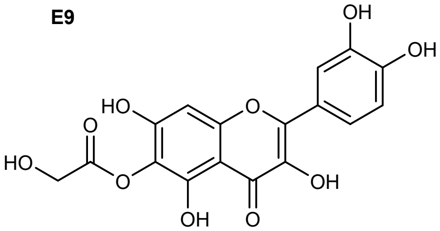

E10 (R)

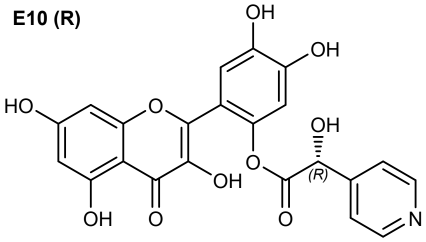

E10 (S)

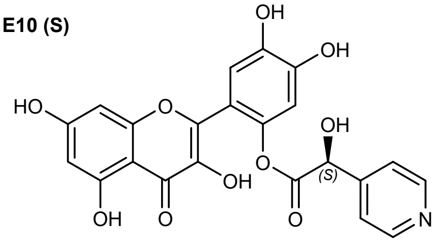

E11

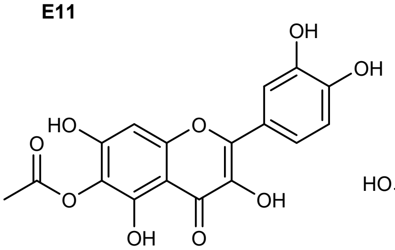

E12

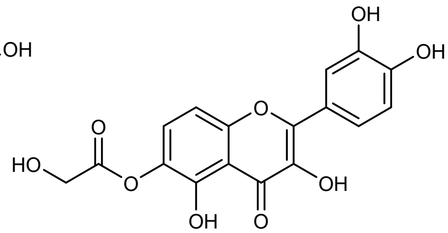

E13

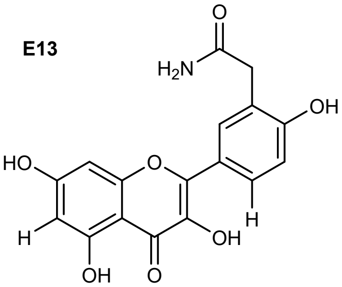

Supplementary Figure 1. Chemical structures of all compounds assessed by the computational docking experiment. Series (A) based around thiourea, (B)&(C) 2-MA, (D) quercetin, and (E) quercetin and 2-MA. Compounds B17 (R) and C2 (R), B17 (S) and C2 (S) are the same structure. Compounds were drawn using ChemDraw 19.1.1.21 (PerkinElmer Informatics, Waltham, Massachusetts, US).

Supplementary Table 1. Description of the compounds with docking scores and manually predicted contacts. Compounds are separated by series and ranked according to docking score.

Code	R groups							Docking Score (LF dG)	Manually predicted number of contacts
	R_1	R_2	R_3	R_4	R_5	R_6	R_7		
Series A									
A9	COOH	CH_2(pyridin-3-yl)	-	-	-	-	-	-9.652	9
A3	COOH	H	-	-	-	-	-	-9.007	6
A4	CSSH	H	-	-	-	-	-	-8.714	8
A12	benzene	CH_2(pyridin-3-yl)	-	-	-	-	-	-8.209	4
A11	CH_2(pyridin-3-yl)	CH_2(pyridin-3-yl)	-	-	-	-	-	-7.213	6
A7	pyridin-3-yl	H	-	-	-	-	-	-7.017	7
A13	isopropyl	CH_2(pyridin-3-yl)	-	-	-	-	-	-6.945	5
A6	CH_2(benzene)	H	-	-	-	-	-	-6.581	3
A10	H	CH_2(pyridin-3-yl)	-	-	-	-	-	-6.374	9
A5	benzene	H	-	-	-	-	-	-6.102	3
A16	COCH3	H	-	-	-	-	-	-5.925	5
A8	3-methyl pyridin-4-yl	H	-	-	-	-	-	-5.759	5
A15	isopropyl	H	-	-	-	-	-	-5.671	4
A14	$COCH_3$	CH_2(pyridin-3-yl)	-	-	-	-	-	-5.56	8
A1	Me	H	-	-	-	-	-	-5.419	4
A2	OH	H	-	-	-	-	-	-5.05	5
Thio-urea	H	H	-	-	-	-	-	-4.711	4
Series B									
B17 (R)	pyridin-4-yl	pyridin-4-yl	-	-	-	-	-	-9.321	6
B11 (S)	pyridin-4-yl	H	-	-	-	-	-	-8.852	7
B18	2 x pyridin-4-yl	pyridin-4-yl	-	-	-	-	-	-8.737	4
B9 (R)	pyridin-2-yl	H	-	-	-	-	-	-8.501	7
B17	pyridin-4-	pyridin-4-	-	-	-	-	-	-8.092	5

(S)	yl	yl							
B10 (R)	pyridin-3-yl	H	-	-	-	-	-	-7.949	8
B6 (R)	H	CH(OH)Me	-	-	-	-	-	-7.73	8
B11 (R)	pyridin-4-yl	H	-	-	-	-	-	-7.675	8
B16	H	pyridin-4-yl	-	-	-	-	-	-7.395	8
B7 (S,R)	OH	CH_2OH	-	-	-	-	-	-7.35	12
B10 (S)	pyridin-3-yl	H	-	-	-	-	-	-7.237	9
B8 (S)	benzene	H	-	-	-	-	-	-7.229	5
B2	SH	H	-	-	-	-	-	-7.194	6
B7 (R,S)	OH	CH_2OH	-	-	-	-	-	-7.152	9
B7 (R,R)	OH	CH_2OH	-	-	-	-	-	-7.128	12
B6 (S)	H	CH(OH)Me	-	-	-	-	-	-6.959	13
B7 (S,S)	OH	CH_2OH	-	-	-	-	-	-6.937	15
B9 (S)	pyridin-2-yl	H	-	-	-	-	-	-6.867	11
B13	H	benzene	-	-	-	-	-	-6.483	4
B5	H	Et	-	-	-	-	-	-6.298	6
B12	H	CH_2OH	-	-	-	-	-	-6.288	10
B15	H	pyridin-3-yl	-	-	-	-	-	-6.279	11
B4	H	Me	-	-	-	-	-	-5.871	6
B3 (S)	OH	H	-	-	-	-	-	-5.765	6
B3 (R)	OH	H	-	-	-	-	-	-5.755	8
B1 (2-MA)	H	H	-	-	-	-	-	-5.611	8
B14	H	pyridin-2-yl	-	-	-	-	-	-5.316	8
B8 (R)	benzene	H	-	-	-	-	-	-4.702	5
C10 (R)	$CH(OH)_2$	pyridin-4-yl	pyridin-4-yl	-	-	-	-	-10.195	11
Series C									
C7 (R)	pyridin-4-yl	pyridin-4-yl	pyridin-4-yl	-	-	-	-	-9.761	8
C7 (S)	pyridin-4-yl	pyridin-4-yl	pyridin-4-yl	-	-	-	-	-9.617	6
C2 (R)	H	pyridin-4-yl	pyridin-4-yl	-	-	-	-	-9.288	5
C9 (S)	CH_2OH	pyridin-4-yl	pyridin-4-yl	-	-	-	-	-9.209	9
C6 (S)	isopropyl	pyridin-4-yl	pyridin-4-yl	-	-	-	-	-9.158	7
C8 (R)	OH	pyridin-4-yl	pyridin-4-yl	-	-	-	-	-9.088	7
C9 (R)	CH_2OH	pyridin-4-yl	pyridin-4-yl	-	-	-	-	-8.955	7
C6 (R)	isopropyl	pyridin-4-yl	pyridin-4-yl	-	-	-	-	-8.942	5
C8 (S)	OH	pyridin-4-	pyridin-4-	-	-	-	-	-8.888	9

		yl	yl						
C3 (R)	Et	pyridin-4-yl	pyridin-4-yl	-	-	-	-	-8.841	7
C2 (S)	H	pyridin-4-yl	pyridin-4-yl	-	-	-	-	-8.692	3
C1 (R)	Me	pyridin-4-yl	pyridin-4-yl	-	-	-	-	-8.594	8
C16	pyridin-4-yl	H	pyridin-4-yl	-	-	-	-	-8.579	3
C24 (S)*	$CH(OH)_2$	pyridin-4-yl	pyridin-4-yl	-	-	-	-	-8.526	14
C5 (S)	CH_2(benzene)	pyridin-4-yl	pyridin-4-yl	-	-	-	-	-8.436	9
C23 (R)*	pyridin-4-yl	pyridin-4-yl	pyridin-4-yl	-	-	-	-	-8.311	7
C4 (S)	benzene	pyridin-4-yl	pyridin-4-yl	-	-	-	-	-8.04	8
C23 (S)*	pyridin-4-yl	pyridin-4-yl	pyridin-4-yl	-	-	-	-	-7.994	8
C10 (S)	$CH(OH)_2$	pyridin-4-yl	pyridin-4-yl	-	-	-	-	-7.974	11
C24 (R)*	$CH(OH)_2$	pyridin-4-yl	pyridin-4-yl	-	-	-	-	-7.97	13
C3 (S)	Et	pyridin-4-yl	pyridin-4-yl	-	-	-	-	-7.627	10
C5 (R)	CH_2(benzene)	pyridin-4-yl	pyridin-4-yl	-	-	-	-	-7.54	8
C14	CH_2(benzene)	H	pyridin-4-yl	-	-	-	-	-7.497	4
C13	benzene	H	pyridin-4-yl	-	-	-	-	-7.47	2
C20	CH_2(benzene)	H	H	-	-	-	-	-7.371	5
C1 (S)	Me	pyridin-4-yl	pyridin-4-yl	-	-	-	-	-7.19	8
C4 (R)	benzene	pyridin-4-yl	pyridin-4-yl	-	-	-	-	-7.077	5
C11	Me	H	pyridin-4-yl	-	-	-	-	-7.014	9
C22 (R)*	CH_2(benzene)	pyridin-4-yl	pyridin-4-yl	-	-	-	-	-6.981	7
C19	benzene	H	H	-	-	-	-	-6.92	6
C15	isopropyl	H	pyridin-4-yl	-	-	-	-	-6.819	8
C12	Et	H	pyridin-4-yl	-	-	-	-	-6.686	5
C22 (S)*	CH_2(benzene)	pyridin-4-yl	pyridin-4-yl	-	-	-	-	-6.409	7
C21*	benzene	pyridin-4-yl	pyridin-4-yl	-	-	-	-	-6.235	6
C18	Et	H	H	-	-	-	-	-6.213	8
C17	Me	H	H	-	-	-	-	-5.901	6
2-MA	H	H	H	-	-	-	-	-5.758	7
Series D									
Diii2	C	C	C	N	-	-	-	-11.171	12
Diii3	C	N	C	C	-	-	-	-10.631	16
Diii1	C	C	N	C	-	-	-	-10.189	14
Diii4	N	N	C	C	-	-	-	-9.865	14

Div7	C	C	C	N	H	OH	H	-9.716	7
Dv2	y	-	-	-	-	-	-	-9.554	10
Dii2	NH_2	-	-	-	-	-	-	-9.197	10
Div6	C	C	C	N	H	H	OH	-9.163	10
Di2	OH	OH						-9.15	11
Div8	C	C	C	N	OH	H	H	-9.11	9
Dv1	x	-	-	-	-	-	-	-9.103	6
Di1	OH	H	-	-	-	-	-	-8.897	10
Dv3	z	-	-	-	-	-	-	-8.836	8
Dii1	OH	-	-	-	-	-	-	-8.722	15
Div9	C	C	C	N	H	NH_2	H	-8.678	9
Div3	C	N	C	C	H	-	H	-8.486	7
Div5	C	C	C	N	H	H	H	-8.313	7
Div4	C	C	N	C	H	H	-	-8.113	8
Div2	N	C	C	C	-	H	H	-8.006	8
Div1	C	C	C	C	H	H	H	-7.88	6
Series E									
E5 (S)	OH	H	OH	OCOCSH(pyridin-4-yl)	OH	-	-	-12.902	11
E5 (R)	OH	H	OH	OCOCSH(pyridin-4-yl)	OH	-	-	-11.943	11
E10 (S)	OH	$OCOCH_3$	OH	OCOCOH(pyridin-4-yl)	OH	-	-	-11.71	11
E3	OH	H	OH	$OCOCH_2SH$	OH	-	-	-11.215	11
E2	OH	H	$OCOCH_2SH$	H	OH	-	-	-11.095	9
E14	OH	H	OH	CH_2CONH_2	OH	-	-	-10.252	10
E7	OH	H	OH	$OCOCH_2OH$	OH	-	-	-10.237	10
E10 (R)	OH	$OCOCH_3$	OH	OCOCOH(pyridin-4-yl)	OH	-	-	-10.145	11
E1	OH	H	OH	H	$OCOCH_2SH$	-	-	-9.648	7
E17	OH	H	OH	CH_2CHO	OH	-	-	-9.611	10
E8	OH	H	OH	$OCOCH_3$	OH	-	-	-9.549	10
E13	OH	H	OH	H	CH_2CONH_2	-	-	-9.157	10
E6	OH	H	$OCOCH_2OH$	H	OH	-	-	-9.097	11
E11	OH	$OCOCH_2OH$	OH	H	OH	-	-	-8.754	8
E12	H	H	OH	H	OH	-	-	-8.601	11
E18	H	CH_2CONH_2	OH	H	OH	-	-	-8.6	11
E9	OH	$OCOCH_2OH$	OH	H	OH	-	-	-8.491	11
E16	OH	CH_2CONH_2	OH	H	OH	-	-	-8.403	9
E4	OH	$OCOCH_2SH$	OH	H	OH	-	-	-7.836	9

E15	OH	H	CH_2CONH_2	H	H	-	-	-7.704	7

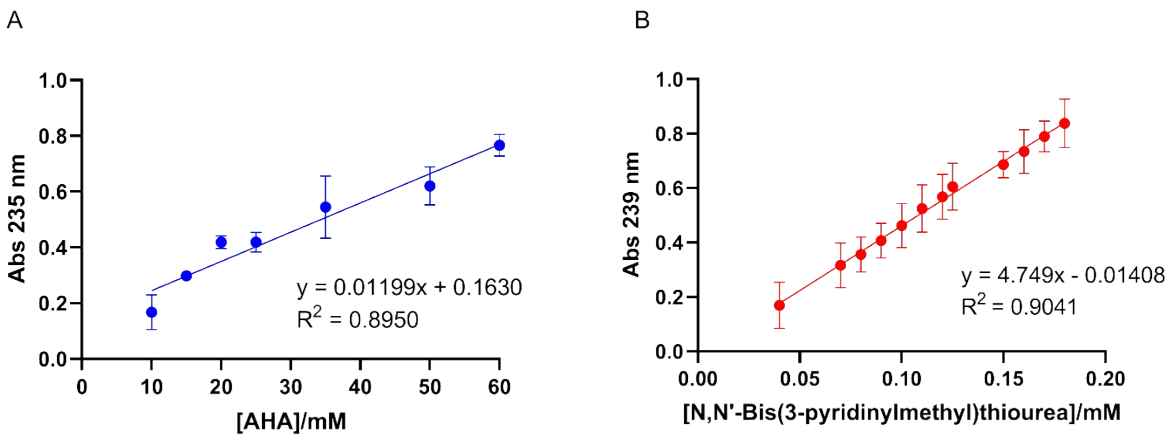

Supplementary Figure 2. Calibration curves of AHA (A) and N,N'-Bis(3-pyridinylmethyl)thiourea (A11) (B), determined using UV-vis spectroscopy for quantifying release through the Biomodics catheter's balloon.

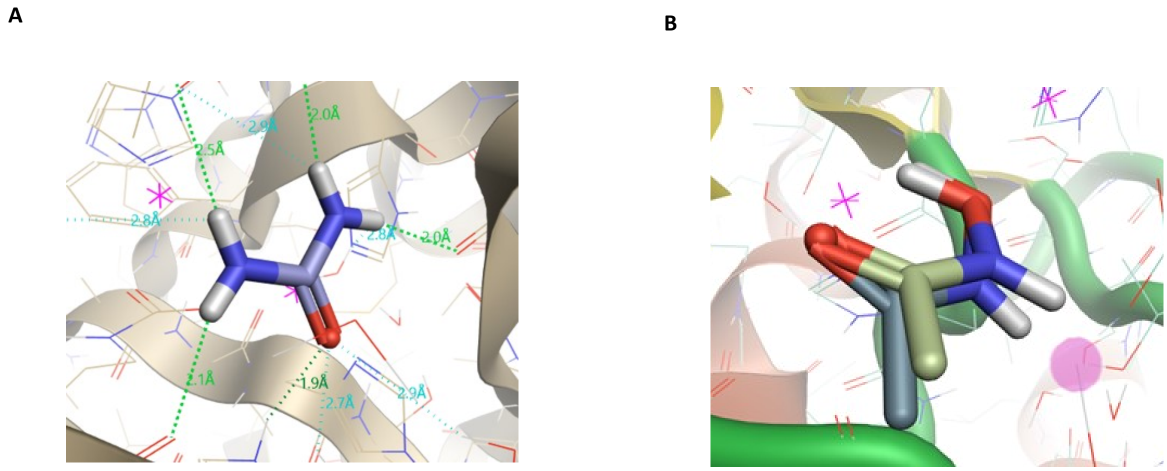

Supplementary Figure 3. A. molecular docking of urea, **B.** AHA, into the active site of *S. pasteruii* urease, the top compound is the crystallized AHA and the bottom is the docked ligand AHA, RMSD = 0.977 Å. Pink asterixis indicate Ni ions in the center of the urease, green dotted lines show the distance between the compound and the amino acids within the active site. Molecules docked with Cresset, Flare v. 4.0.2. Images generated using Flare™ from Cresset®.

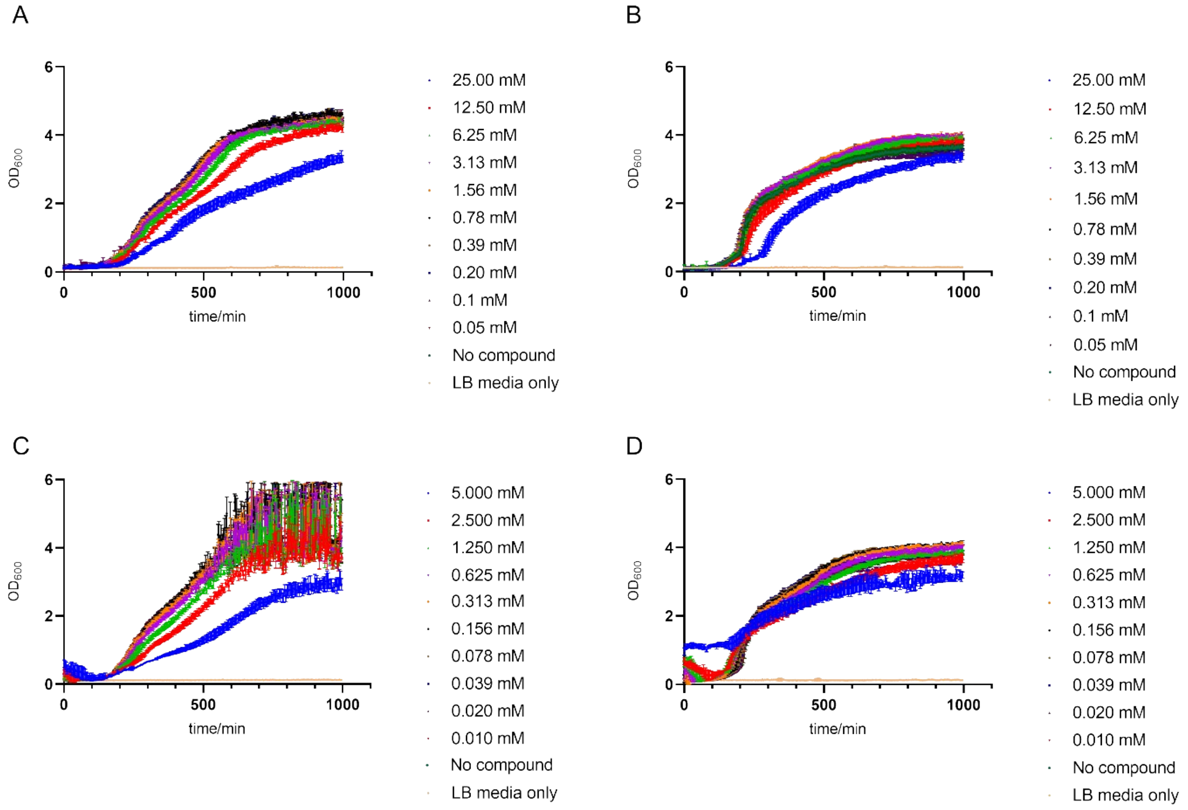

Supplementary Figure 4. Minimum inhibition growth curves. **A.** *P. mirabilis* in the grown in varying concentrations of AHA. **B.** *E. coli* with AHA. **C.** *P. mirabilis* with Bis-TU (A11). **D.** *E. coli* with Bis-TU (A11). The highest concentration of all compounds contained 2.5% DMSO which was diluted further during compound dilutions. The experiment was conducted with three biological repeats, error bars represent standard deviation. Graphs were drawn using GraphPad Prism v.9.4.1.

Supplementary Table 2. MIC ranges for AHA and Bis-TU (A11) in *P. mirabilis* and *E. coli*.

Bacterial species	[AHA]/ mM	[Bis-TU]/mM
P. mirabilis B4	3.13 – 25.0	1.25 – 5.00
E. coli NSM59	12.5 – 25.0	1.25 – 5.00

Supplementary Table 3. IC50 fitting data

Data fitted to:

$$\% \, Urease \; activity = \frac{A1 + (A2 - A1)}{1 + 10^{(logXo - X)}.P}$$

a. *Proteus mirabilis* whole cell

**Model**	Dose Resp					
**Equation**	y = A1 + (A2-A1)/(1 + 10^((LOGx0-x)*p))					
**Plot**	**AHA**	**A5**	**Quercetin**	**A6**	**A11**	**2-MA**
**A1**	1.73858 ± 5.56086	-2833.55254 ± 571357.59569	-6.87402 ± 9.90633	5.56987 ± 6.7131	-6.89555 ± 4.6294	
**A2**	91.30088 ± 2.97347	141.84726 ± 812.39328	68.52898 ± 7.38724	78.71291 ± 4.0369	85.90046 ± 7.52903	
**LOGx0**	-4.43621 ± 0.18293	-5.015 ± 0.11323	-6.06228 ± 0.30034	-4.64408 ± 0.17038	-5.85143 ± 0.25846	
**Reduced Chi-Sqr**	2.92888	3.347	2.64774	88.59753	1.32268	
**Adj. R-Square**	0.97678	0.986	0.9453	0.97726	0.98853	
**Model**	Dose Resp					
